# Postseptal Weight Placement for Paralytic Lagophthalmos

**Published:** 2016-06-22

**Authors:** Adam Gendy, Paul J. Therattil, Adam M. Feintisch, Edward S. Lee

**Affiliations:** Division of Plastic and Reconstructive Surgery, Department of Surgery, Rutgers New Jersey Medical School, Newark

**Keywords:** facial palsy, lagophthalmos, gold weight, lid loading, postseptal placement

**Figure:**
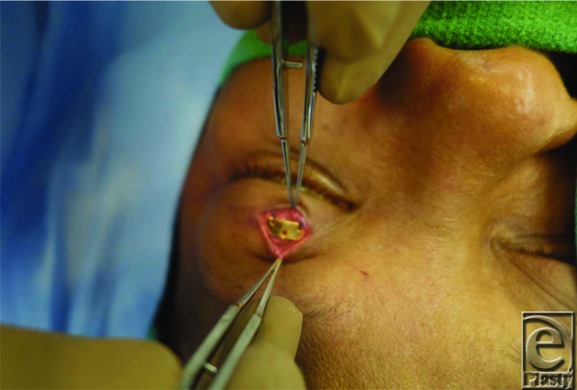


## DESCRIPTION

A 56 year-old female had resection of an invasive parotid tumor infiltrating the facial nerve. The facial nerve was sacrificed during the resection. She presented with the inability to close her left eye completely 1 week status-post resection. On examination, the patient had approximately 5 mm of sclera exposed with attempted eye closure.

## QUESTIONS

1. **What are the surgical indications for gold weight placement?**
2. **What are the complications associated with gold weight placement and how can they be avoided?**
3. **How does the dissection for postseptal gold weight placement differ from traditional placement?**
4. **What are the advantages and disadvantages associated with postseptal placement of gold weights?**

## DISCUSSION

One of the major sequelae of facial nerve palsy is lagophthalmos, or the inability to close the eye completely. In our case, injury to the facial nerve resulted in paralysis of the orbicularis oculi muscle, which is responsible for promoting eyelid closure. As a result, some amount of the corneal surface area remains exposed with attempted eye closure, which places the eye at risk of developing corneal dryness, abrasions, keratitis, ulcerations, and even eventual blindness. While there are several temporary interventions for the management of paralytic lagophthalmos, lid loading with gold weight placement remains a mainstay of definitive, long-term surgical therapy in cases where the functional recovery of the facial nerve is likely to be delayed or incomplete.

Albeit effective, the insertion of weighted gold implants at the upper eyelid carries with it several potential complications including infection (8%),^1^ allergic reaction to gold (1%),^2^ visibility of the implant underneath the skin (31%),^3^ migration of the implant within the eyelid (2.6%),^3^ extrusion (43%),^4^ residual lagophthalmos (30%),^5^ or ptosis (12%).^3^ While some of these complications can be managed simply by exchanging the implant, migration and extrusion of the implant are serious complications that are difficult to predict and avoid. The rate of migration of weighted gold implants ranges from 0.0% to 2.6%,^3^ whereas the rate of extrusion ranges from 5.0% to 50.0%.^6^ The rate of revision surgery to correct complications from gold weight placement is 8.0% to 14.0%.^7^ Recommendations both to prevent migration and extrusion and to decrease the visibility of the implant include anchoring the implant to the underlying tarsal plate with sutures,^4^ insetting the weight further away from the lid margin,^3^ and even advancing the levator aponeurosis over the implant and suturing it inferiorly to the tarsal plate.^8^

**Figure 1:**
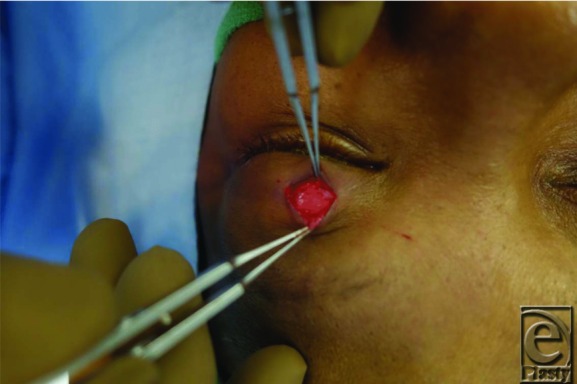
Intraoperative view of the orbital septum of the upper eyelid.

**Figure 2:**
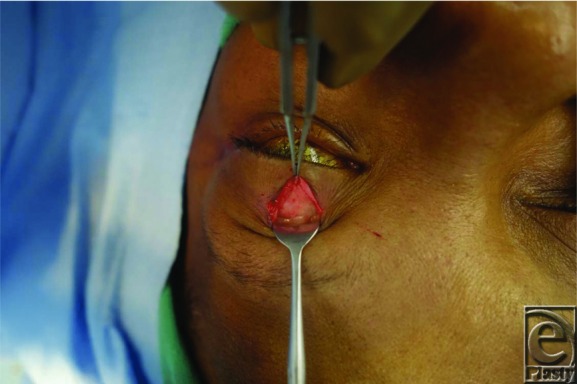
Intraoperative view of the levator aponeurosis and postseptal fat (deep to the retractor) after opening the orbital septum.

**Figure 3:**
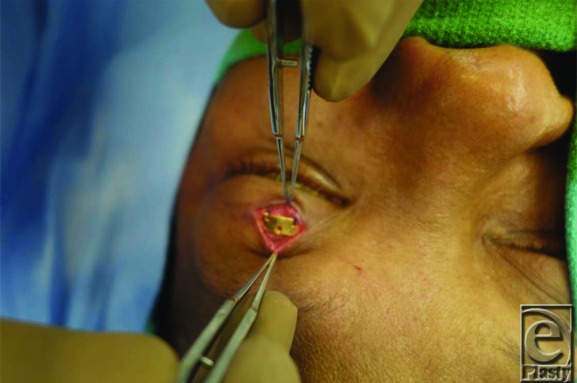
Intraoperative view of postseptal gold weight placement with weight sutured to the levator aponeurosis just above the tarsal plate.

Traditional placement of gold weights within the upper eyelid is in a submuscular pocket, deep into the orbicularis oculi muscle and superficial to the anterior surface of the tarsal plate. The implant is then placed deep into the orbicularis oculi muscle, after which the muscle and the overlying skin are redraped over the weight for coverage. An alternative technique involves placement of the implant deep into the orbital septum, which may offer better coverage of the implant. In this procedure, the septum is accessed approximately 2 to 3 mm above the superior border of the tarsal plate (Fig 1). The septum is incised and the postseptal fat is retracted superiorly to visualize the underlying levator aponeurosis and muscle (Fig 2). The implant is then sutured to the levator, later to be covered by the postseptal fat along with the repaired orbital septum and the overlying orbicularis muscle (Fig 3).

**Figure 4:**
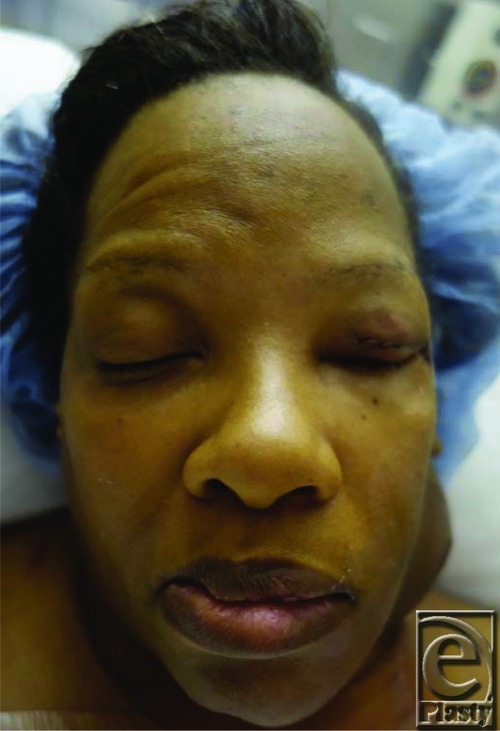
Patient demonstrating complete resolution of lagophthalmos after gold weight placement.

The postseptal approach is advantageous in that it addresses several of the feared complications associated with the traditional placement of gold weights: the addition of another layer of tissue over the implant, in theory, decreases the likelihood of the implant extrusion and may help decrease the visibility of the weight through the skin. The primary disadvantage of this technique is that the implant sits higher up on the convex surface of the upper lid, which results in a decrease in the downward vector force produced. This can be overcome, however, simply by employing a heavier weight to generate the desired inferior vector force. To date, there have been no documented complications associated with the postseptal technique, with the longest follow-up period reported in the literature being 4 years.^5^ Our patient underwent postseptal placement of a 1-g gold weight and postoperatively had complete resolution of her lagophthalmos without complication (Fig 4).
